# Biological oscillations for learning walking coordination: dynamic recurrent neural network functionally models physiological central pattern generator

**DOI:** 10.3389/fncom.2013.00070

**Published:** 2013-05-29

**Authors:** Thomas Hoellinger, Mathieu Petieau, Matthieu Duvinage, Thierry Castermans, Karthik Seetharaman, Ana-Maria Cebolla, Ana Bengoetxea, Yuri Ivanenko, Bernard Dan, Guy Cheron

**Affiliations:** ^1^Laboratory of Neurophysiology and Movement Biomechanics, CP601, ULB Neuroscience Institute, Université Libre de BruxellesBrussels, Belgium; ^2^TCTS Lab, Faculty of Engineering, Université de MonsMons, Belgium; ^3^Laboratory of Neuromotor Physiology, Fondazione Santa LuciaRome, Italy; ^4^Laboratory of Electrophysiology, Université de MonsMons, Belgium

**Keywords:** central pattern generator (CPG), human locomotion, biological oscillations, dynamical recurrent neural network (DRNN), kinematics, neurophysiology of walking

## Abstract

The existence of dedicated neuronal modules such as those organized in the cerebral cortex, thalamus, basal ganglia, cerebellum, or spinal cord raises the question of how these functional modules are coordinated for appropriate motor behavior. Study of human locomotion offers an interesting field for addressing this central question. The coordination of the elevation of the 3 leg segments under a planar covariation rule (Borghese et al., 1996) was recently modeled (Barliya et al., 2009) by phase-adjusted simple oscillators shedding new light on the understanding of the central pattern generator (CPG) processing relevant oscillation signals. We describe the use of a dynamic recurrent neural network (DRNN) mimicking the natural oscillatory behavior of human locomotion for reproducing the planar covariation rule in both legs at different walking speeds. Neural network learning was based on sinusoid signals integrating frequency and amplitude features of the first three harmonics of the sagittal elevation angles of the thigh, shank, and foot of each lower limb. We verified the biological plausibility of the neural networks. Best results were obtained with oscillations extracted from the first three harmonics in comparison to oscillations outside the harmonic frequency peaks. Physiological replication steadily increased with the number of neuronal units from 1 to 80, where similarity index reached 0.99. Analysis of synaptic weighting showed that the proportion of inhibitory connections consistently increased with the number of neuronal units in the DRNN. This emerging property in the artificial neural networks resonates with recent advances in neurophysiology of inhibitory neurons that are involved in central nervous system oscillatory activities. The main message of this study is that this type of DRNN may offer a useful model of physiological central pattern generator for gaining insights in basic research and developing clinical applications.

## Introduction

Neuronal modules profoundly influence many aspects of motor behavior. However, little is currently known about the control mechanisms that allow the coordination of these modular entities. In this theoretical context, human locomotion is a challenging movement because of the numerous neuroanatomical modules implicated in the different aspects of whole body movement, ranging from the cyclic propulsion of the limb to balance control. In spite of these intricate movement components and neuronal modules involved, it has been proposed that the overall control can be realized by reducing the number of degrees of freedom of the system into global variables (Bernstein, 1967; Lacquaniti et al., 1999, 2002; Flash and Hochner, 2005; Latash, 2008). The fact that the elevation angles of the three main lower limb segments are coordinated during gait within a covariation plane (Borghese et al., 1996), forming an elliptic loop corroborates the idea that control of locomotion is also submitted to the general law of reducing variables (Barliya et al., 2009). This general law is also applicable for different walking speeds (Bianchi et al., 1998), for forward and backward directions (Grasso et al., 1998), rectilinear or curvilinear walking paths (Courtine and Schieppati, 2004), walking with stilts (Dominici et al., 2009; Leurs et al., 2011), or with a transfemoral prosthesis walk (Leurs et al., 2012), with different levels of body weight unloading (Ivanenko et al., 2002) and for running (Ivanenko et al., 2007). Notably, this inter-segmental coordination is not present in newly walking toddlers (Cheron et al., 2001a,b; Ivanenko et al., 2005), but covariation planarity rapidly emerges over the first few days of independent walking experience, while the shape of the loop and plane orientation “mature” gradually over several years. This evolution indicates that the attractor plane and the shape of the loop are neurophysiologically defined, rather than being imposed by biomechanical constraints (see Hicheur et al., 2006; and Ivanenko et al., 2008 for discussion). More recently, the developmental study of this complex behavior in a new born baby (Dominici et al., 2011) has permitted revisiting the concept of locomotor modules coding for the control of movement primitives.

This modular approach raises the question of the dynamic coordination of modules in the context of oscillatory properties of neuronal ensembles. Indeed, the dynamical structure of these modules must logically obey a common principle for movement generation: the production of oscillatory activity. Although this principle is well accepted in case of the rhythmic nature of locomotion (Georgopoulos and Grillner, 1989; Grillner, 2006), the recent study of Churchland et al. (2012) surprisingly demonstrates that non-periodic movements such as reaching are also generated by neuronal oscillation. This means that there is a strong possibility that the spinal modules organized in a central pattern generator (CPG) could be dynamically controlled by cortical and/or supraspinal oscillations. Interestingly, Barliya et al. (2009) recently modeled the time course of elevation angles of the three lower limb segments in terms of simple oscillators coupled with appropriate time shifts reproducing the orientation of the plane and their elliptical shape. The oscillators were obtained by taking, after Fourier transform, the first three harmonics of the elevation angle kinematics. Each of these oscillators could be interpreted in term of oscillatory module acting in such a way that the orientation of the plane and the elliptic shape of the coordination are conserved. It could thus be possible that oscillatory signals derived from these harmonics are used for activating CPG modules.

The existence of CPG in the spinal cord in mammals has been proposed a century ago (Brown, 1911). In essence, it represents a group of neurons acting as a network to produce coordinated patterns of rhythmic activity. New evidence has shown the presence of CPG in the spinal cord in humans (Calancie et al., 1994; Bussel et al., 1996). The characteristics of such CPG modules are their adaptability and robustness that lead to the production of different gait patterns adapted to their current environmental context. For example, young infants (less than 1-year old) are already able to walk over a split-belt treadmill with different types of coupling (Yang et al., 2005). Some of them were even able to walk in opposite directions. Mimicking physiology, the robotic and neuroscientific community developed artificial CPGs that are commonly used to animate robots from multi-legged insect-like to humanoids (for a review see Ijspeert, 2008). In their pioneering work in the cat, Patla et al. (1985) proposed an analytic model of limb locomotor pattern generator based on recorded muscle activity induced by electrical stimulation over the mesencephalic locomotor region (MLR) in the decerebrate cat. In this model, locomotor like patterns of six muscles resulted from six independent oscillators with dedicated parameters. These oscillators were reduced to simple sine and cosine functions fed by a tonic input. Since then, different methods have been used from coupled non-linear oscillators (Ijspeert et al., 2007; Duvinage et al., 2011, 2012a), to highly detailed biophysics of small groups of interconnected neurons (Hellgren et al., 1992) and rhythm genesis of larger groups of neurons (Wadden et al., 1997) mainly in animal models. While human locomotion has often been reproduced computationally in the robotic field using equations that are numerically integrated (Righetti et al., 2005; Righetti and Ijspeert, 2006; Ceccato et al., 2009; Duvinage et al., 2011, 2012a), few methods involving neuron modeling for human gait generation have been studied so far. Among them Prentice and coauthors (1998, 2001) have successfully transformed fundamental timing signals (sine and cosine inputs) into individual muscles activation bursts related to gait locomotion at different speeds using a feedforward neural network. Our group used the electromyographic (EMG) signals of the lower limb muscles as input for a dynamic recurrent neural network (DRNN) producing as output the lower kinematics during locomotion (Cheron et al., 2003, 2012). However, the possibility to produce the motion of the lower limb segments by means of oscillations derived from the three harmonics of the Fourier transform of walking kinematics has not yet been assessed by means of the same DRNN. We show here that after learning based on different walking velocities, the DRNN is able to reproduce the lower limb kinematics of both legs. The DRNN can also generalize to the unlearned walking velocities. The analysis of the required network structure (e.g., number of units, distribution of time constant, and synaptic sign) provides a basis for the discussion about the constraints required for the elaboration of a CPG.

## Methods

### Experimental setup

**Figure 1:**
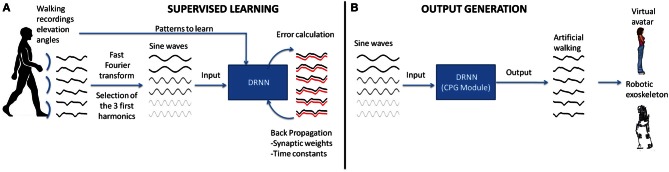
**Realization of a CPG module based on experimental recordings of human walking. (A)** In the first step, the elevation angles of the thigh, shank, foot of the two legs are transformed in the time frequency domain using fast Fourier transform (FFT). Then the signal characteristics are computed for the first three harmonics and back-transformed into temporal space using sine waves formulation. These data are fed as input to the DRNN learning the transformation in kinematic signals (elevation angles). **(B)** A successful learning will permit the DRNN to predict kinematics based on sine wave inputs only. These output signals can produce biologically plausible walking patterns in a virtual reality avatar or an actual robotic exoskeleton.

Seven healthy subjects aged from 25 to 35 years (mean age: 28 years) participated in this experiment. The protocol consisted of walking on a treadmill at 11 different velocities [from 1 (0.28 m/s) to 6 km/h (1.67 m/s) stepped by 0.5 km/h (0.14 m/s)] leading to 11 trials per subject (total of 77 trials over all subjects). Whole body kinematics was recorded using Vicon system with six infrared Bonita cameras recording at 100 Hz during 40 s for each trial. The tracking consisted of recording 29 markers placed over the whole body. The position of the markers in an orthogonal reference was computed using a custom biomechanical model. From the position of the markers of both legs, the kinematic (elevation) angles relative to the vertical plane of the laboratory have been calculated bilaterally for thighs, shanks, and feet. In this study we performed two experiments. The first one, “proof of concept,” was done to ascertain the feasibility of a DRNN to learn elevation angles from pure sine waves. This part only includes supervised learning on a single pattern (Figure 1A). The second experiment was performed in order to study the possibility to learn multiple patterns of walking (i.e., velocities) and to predict kinematics from unlearned patterns (Figure 1B). After that, the DRNN structures were analyzed.

### Dynamical recurrent neuronal network

The DRNN is capable of modeling time-varying input–outputs and has varying weights as well as varying time constants for the artificial neurons (Pearlmutter, 1990). The adaptive time constants make it dynamic (Draye et al., 1996). The DRNN is governed by the following equations:

(1) $$T_{i}\frac{dy_{i}}{dt}=-y_{i}+F(x_{i})+I_{i}$$

where *F*(*a*) is the squashing function *F*(*a*) = (1 + *e*^−*a*^)^−1^, *y*_*i*_ is the state or activation level of unit *i*, *I*_*i*_, is an external input (or bias), and *x*_*i*_ is given by:

(2) $$x_{i}=\sum_{j}w_{ij}y_{i}$$

which is the propagation equation of the network (*x*_*i*_ is called the total or effective input of the neuron *i*, *w*_*ij*_ is the synaptic weight between units *i* and *j*). The time constants *T*_*i*_ will act like a relaxation process. In order to make the temporal behavior of the network explicit, an error function is defined as:

(3) $$E=\int_{t_{0}}^{t_{1}}q(y(t),t)dt$$

where *t*_0_ and *t*_1_ give the time interval during which the correction process occurs. The function *q*(*y*(*t*), *t*) is the cost function at time *t* which depends on the vector of the neuron activations *y* and on time. We then introduce new variables *p*_*i*_ (called adjoint variables) that will be determined by the following system of differential equations:

(4) $$\frac{dp_{i}}{dt}=\frac{1}{T_{i}}p_{i}-e_{i}-\sum_{j}\frac{1}{T_{i}}w_{ij}F^{\prime }(x_{j})p_{j}$$

with boundary conditions *p*_*i*_(*t*_1_) = 0. After the introduction of these new variables, we can derive the learning equations:

(5) $$\frac{\delta E}{\delta w_{ij}}=\frac{1}{T_{i}}\int_{t_{0}}^{t_{1}}y_{i}F^{\prime }(x_{j})p_{j}dt$$

(6) $$\frac{\delta E}{\delta T_{i}}=\frac{1}{T_{i}}\int_{t_{0}}^{t_{1}}p_{i}\frac{dy_{i}}{dt}$$

**Figure 2:**
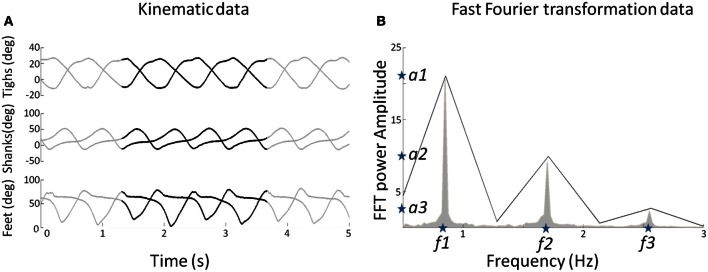
**(A)** Elevation angles (in degree) of the three segments (thigh, shank, and foot) for each leg as a function of time for a subject walking at 3 km/h (0.83 m/s). The whole pattern is presented for duration of 5 s. The black lines represent a pattern for two gait cycles used to determine the FFT characteristics. **(B)** The mean FFT transformation for six joints for 40 s (in gray) and the two gait cycles (in black). Note that the two gait cycle patterns are selected so as to preserve the FFT characteristics in terms of amplitude and frequency (stars) for the overall pattern of 40 s. These characteristics are used as parameters to generate sine waves as input of the DRNN.

The sinusoid signals derived from the Fast Fourier transform (FFT) kinematic data are sent as input to a DRNN (Figures 1, 2 cf. Experiments 1, 2) that has to learn from these data to produce the kinematics specified as elevation angles (Figure 1A). Successful trainings were also used to produce kinematic patterns from unknown inputs (Figure 1B) aiming to produce walking for multiple purposes, such as virtual avatars or robotic exoskeletons. The training is supervised, involving learning rule adaptations of synaptic weights and time constant of each unit (Draye et al., 1995, 1996). A specific training procedure using Almeida algorithm was used to optimize learning performance (Cheron et al., 2011). The DRNN involves a looping mechanism (fully connected structure) which enables this network to learn and store information (memory). This equips the network with the ability to model complex situations with multiple influences. The DRNN was successfully used to map EMG signals into kinematics during walking (Cheron et al., 2003), for the identification of the triphasic EMG pattern in subjects performing fast elbow flexion movements (Cheron et al., 2007) or to predict specific muscular activity in elite fencers compare to amateurs (Cheron et al., 2011).

### Experiment 1: proof of concept

**Figure 3:**
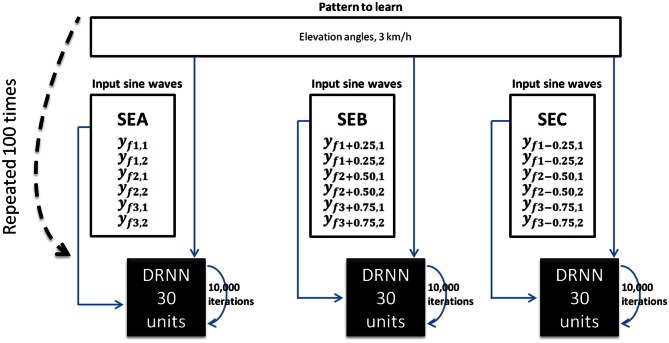
**DRNN learning method for Experiment 1.** For each subject, we trained 100 DRNNs to learn a pattern of kinematic [corresponding to a velocity of 3 km/h (3.83 m/s)] from sine waves (Equations 7 and 8). Three sets of learning were defined as the input differed (SEA, SEB, SEC). The structures of DRNNs were modeled with 30 hidden units for each set of training. Each network iterated 10,000 times to change its synaptic weights and time constants. For each subject and structure we selected the network with the highest *SI* value. For each condition, the design of the network is then processed with 6 inputs, 30 hidden units, and 6 outputs.

DRNN computation has been used to prove that simple sine waves with specified temporal characteristics can be used as input to an artificial neural network to be transformed into elevation angles obtained from kinematic recordings during locomotion (Figure 3).

#### Input: construction of sine waves

As the learning phase of the DRNN is a time-consuming process, we had to select appropriate sample from the whole available data as input. Moreover, even if the kinematics during walking is relatively stable, it may vary too much to feed the DRNN during the learning phase. For these reasons, we extracted two consecutive gait cycles from the 40 s of experimental data recorded in each trial (Figure 2A, black curves). They were chosen so as to be representative in terms of frequency of the whole recording set. Then, to determine the kinematic characteristics of gait, we transformed the data of the lower limb segments elevation angles into the time frequency domain using the Matlab *fft* function to get the FFT power amplitude and their related frequency values of the first three harmonic peaks (Figure 2B). It has been shown previously that the first two Fourier harmonics accounted for approximately 98% of the experimental variance of the thigh, shank, and foot angles (Bianchi et al., 1998). We decided to create sine waves based on the characteristics of the first three harmonics to ensure that the signal proposed as learning input to the DRNN contains enough information to match the desired output precisely.

We extracted the values of the three frequencies (*f*1, *f*2, *f*3) corresponding to the maximal amplitudes (*a*1, *a*2, *a*3). It was verified that *f*2 = 2*f*1 and *f*3 = 3*f*1. We then artificially produced six sinusoidal signals using frequency values as parameters (Equations 7 and 8).

(7) $$y_{fi,1}=\sin(2\pi \times fi\times t)$$

(8) $$y_{fi,2}=\sin(-(2\pi \times fi\times t))$$

(9) For i=1, 2, 3

These six sine waves thus correspond to the frequency characteristics of the kinematic patterns that will be further used as pattern to be learned. For the sake of clarity we called the set of original inputs set SEA (for set of Equations A). Additionally, we produced six new sine waves using the preceding computations (Equations 7 and 8) where *f*1′ = *f*1 + 0.25 Hz and six other sine waves where *f*1″ = *f*1−0.25 Hz, respectively called SEB (for set of Equations B) and SEC (for set of Equations C). Please note that in the latter two cases the original relations *f*2 = 2*f*1 and *f*3 = 3*f*1 were respected and hence *f*2′ = 2*f*1 + 0.50 Hz; *f*3′ = 3*f*1 + 0.75 Hz in the set SEB and *f*2″ = 2*f*1−0.50 Hz; *f*3″ = 3*f*1−0.75 Hz in the set SEC. Three different input sets (SEA, SEB, SEC) were thus defined for learning.

#### Pattern to learn: kinematic data

The pattern to learned corresponds to the elevation angles of the right and left thigh, shank, foot segments in the two gait cycles of a 3 km/h (0.83 m/s) walk, normalized between −1 and 1. The outputs were the same for SEA, SEB, and SEC whereas inputs differed.

#### DRNN learning phase

It was expected that the DRNN would learn how to transform the input to output thanks to its dynamical and recurrent structure of 30 hidden neurons. For each of the seven subjects, the network iterated 10,000 times. This process was repeated 100 times, leading to 100 different networks. At the end of the learning phase, we selected for each subject the network for which the difference between predicted and real kinematics was minimal. This computation was performed by calculating a similarity index (*SI*) (Bengoetxea et al., 2010) defined by the following equation:

(10) $$SI=\frac{\int_{-\frac{Tc}{2}}^{\frac{Tc}{2}}p_{1}(t)p_{2}(t)dt}{{\left[\int_{-\frac{Tc}{2}}^{\frac{Tc}{2}}p_{1}{\left(t\right)}^{2}dt\int_{-\frac{Tc}{2}}^{\frac{Tc}{2}}p_{2}{\left(t\right)}^{2}dt\right]}^{\frac{1}{2}}}$$

where *Tc* is the period of the limit cycle, *p*_1_ and *p*_2_ being synchronized, i.e., the matching between both patterns is based on the maximum of each pattern. Note that if both functions are identical, *SI* = 1. *SI* was calculated with the recorded pattern of elevation angles and the output of the DRNN.

### Experiment 2: multiple pattern learning and prediction

In this experiment sine waves were modulated in frequency and amplitude and transformed into kinematic data using multi-pattern training. The prediction of kinematic pattern from unknown sine wave pattern was also tested.

#### Input

As for the proof of concept methods, we extracted two gait cycles of each trial for multiple velocities (in km/h) (*v* = {1.5, 2.5, 3.5, 4.5, 5.5)}. We transformed the kinematic data into the time-frequency domain to get their frequency (*f*1, *f*2, *f*3) and amplitude (*a*1, *a*2, *a*3) (Figure 2B) parameters using the following set of Equations (10 and 11).

(11) $$y_{v},fi_{v},ai_{v,1}=ai_{v}\times (sin(2\pi \times fi_{v}\times t))$$

(12) $$y_{v},fi_{v},ai_{v,2}=ai_{v}\times (sin(-(2\pi \times fi_{v}\times t)))$$

(13) For i=1, 2, 3

#### Patterns to learn: kinematic data

The patterns to be learned consisted of elevation angles of the right and left thigh, shank, and foot segments corresponding to the two gait cycles, normalized between –1 and 1. These patterns were obtained from recordings at multiple velocities (in km/h) (*v* = {1.5, 2.5, 3.5, 4.5, 5.5}).

#### DRNN training phase

We used the possibility to train the DRNN in a multi-pattern purpose (Bengoetxea et al., 2005). For each subject, the DRNN was trained to match the inputs/outputs patterns corresponding to five different velocities within a single DRNN structure. Hundred networks were made per subject, each of them iterating 50,000 times. This operation was assigned for 1, 2, 3, 4, 5, 6, 7, 8, 9, 10, 20, 30, 40, 50, 60, 70, and 80 hidden units. When this was completed, we selected the best network for each subject and each number of hidden units using the maximal *SI* values.

#### Output prediction

We built sine waves from intermediary pattern velocities (km/h) (*v* = {2, 3, 4, 5}) as explained above. We fed the best DRNN structures previously obtained after the training phases with these unlearned inputs sine waves and analyzed DRNN behavior by calculating the predicted output with experimental data using *SI* values.

### Neuronal properties and connectivity after learning

Introduction of timing allows modeling of more complex frequency behavior, improves the non-linearity effect of the sigmoid function and the memory effect of time delays (Draye et al., 1995). The distribution of the time constant and the synaptic weights between units (Draye et al., 1996) after learning was analyzed after multiple pattern learning and prediction. In particular, we recorded the number of positive and negative connections inside DRNN structures of the best networks. Additionally we studied the distribution of neuronal time-constants.

### Statistical analysis

Statistical analysis was performed using Statistica software (Statsoft, www.statsoft.com). For each test performed and described in the result section, we firstly verify whether the data were distributed normally using Kolmogorov–Smirnov test. When the data were normally distributed we use ANOVA with repeated measures and *post-hoc* Fisher analyses. When it was not possible to use ANOVA we used non-parametric tests such as Friedman ANOVA or sign tests.

### Computations

**Figure 4:**
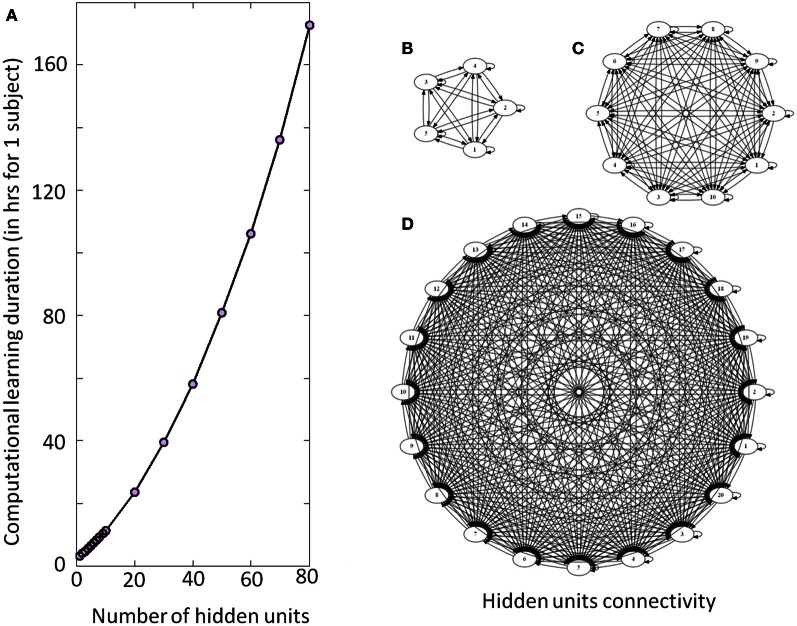
**Illustration of the DRNN structure and computational time. (A)** Computational learning duration for one subject for experiment 2 (in hours). The gray circles represent the estimation of time required per number of hidden units from a recalculation of 5% of the procedure. Please note that the overall process for the seven subjects has taken approximately 200 days to be computed. **(B)**, **(C)**, and **(D)** represent the connectivity of the hidden layer with 5, 10, and 20 neurons, respectively. As the number of neurons increases, the number of connections increases by a factorial multiplication as well as the time required to adjust connection weight and time constant. Please note that only the units of the hidden layers are represented without input or output neurons.

All DRNN computations were performed in the Hydra computing center hosted in ULB (https://cc.ulb.ac.be/hydra/). We allocated 1 node and 10 Gb of memory per computation (i.e., per subject per condition in the Experiment 1, per subject per structure in the Experiment 2). The computations were run in parallel to optimize the learning duration. Afterwards we simulated 5% of the overall experiment in the same conditions to estimate the learning time. The overall duration of the process was obtained by linear interpolation (Figure 4).

## Results

### Experiment 1: proof of concept

**Figure 5:**
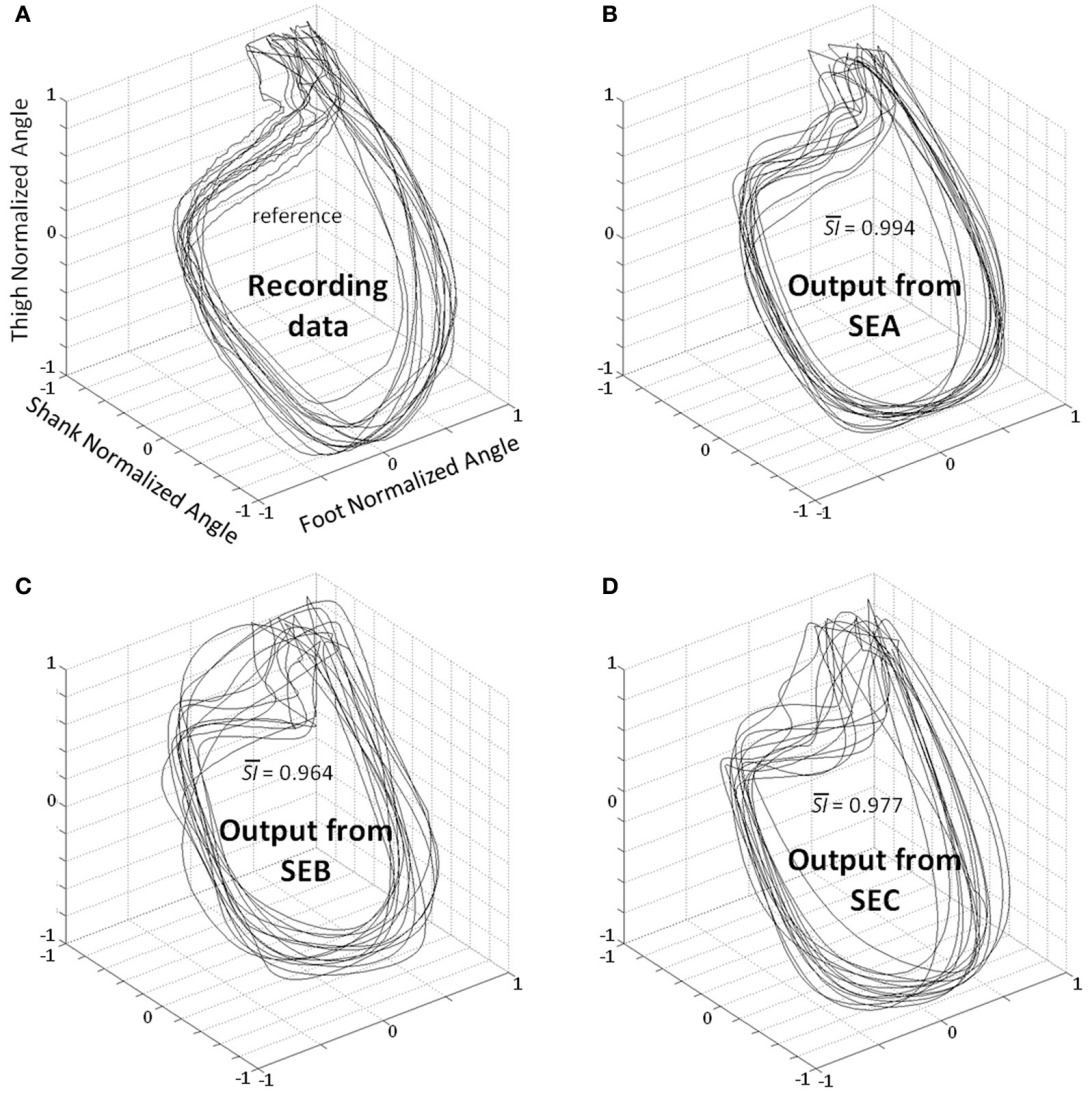
**Planar covariation between normalized thigh, shank, and foot for all participants. (A)** Recording data—the patterns correspond to the real kinematics of 2 gait cycles for all participants. Best kinematic patterns predicted by the DRNN for each subject where SEA **(B)**, SEB **(C)**, and SEC **(D)** were fed as input. $\bar{SI}$ is the average of similarity index value (*SI*) in each condition for all subjects when compared with the recording data.

A statistical test was designed to compare *SI* values from different input types (SEA, SEB, or SEC) (Figures 3,5). The Kolmogorov–Smirnov did not reject the hypothesis that *SI* values were normally distributed (*D* = 1.4414, *p* > 0.20) when analyzing together values of the different inputs (SEA, SEB, or SEC). We used an ANOVA with repeated measures where dependant variable was *SI* and the independent variable chosen was the type of input. The analysis showed an effect of the input frequency in the DRNN prediction (*SI* value) [*H F*_(2, 12)_ = 38.110, *p* = 0.00001]. *Post-hoc* analysis confirmed that *SI* values of original group of unchanged frequency input (SEA) were higher than the 2 modified groups where frequency inputs have changed (SEB and SEC).

### Experiment 2: prediction of intermediary velocities

As we decided to use frequency (*f*1, *f*2, *f*3) and amplitude (*a*1, *a*2, *a*3) characteristics to modulate inputs for multiple learning procedures (Equations 10 and 11), we have verified that there was a statistical significance of these parameters for different velocities. The Kolmogorov–Smirnov test for *f*1 (*D* = 0.14370, *p* < 0.1), *f*2 (*D* = 0.14370, *p* < 0.1), and *f*3 (*D* = 0.14370, *p* < 0.1) was not clear enough to reject the fact their population may follow a normal law. When looking at the distribution, they tend to be normal and it is possible that the significance of the test is due to the weak number of values. According to similar test, the values for parameters *a*1 (*D* = 0.08476, *p* > 0.2), *a*2 (*D* = 0.08758, *p* > 0.2), and *a*3 (*D* = 0.09035, *p* > 0.2) were normally distributed. We then used two ANOVA with repeated measures where the dependent variables were, respectively, the amplitude and frequency values and the within-subject factors were velocity of walking and the specific harmonic (1, 2, or 3).

**Figure 6:**
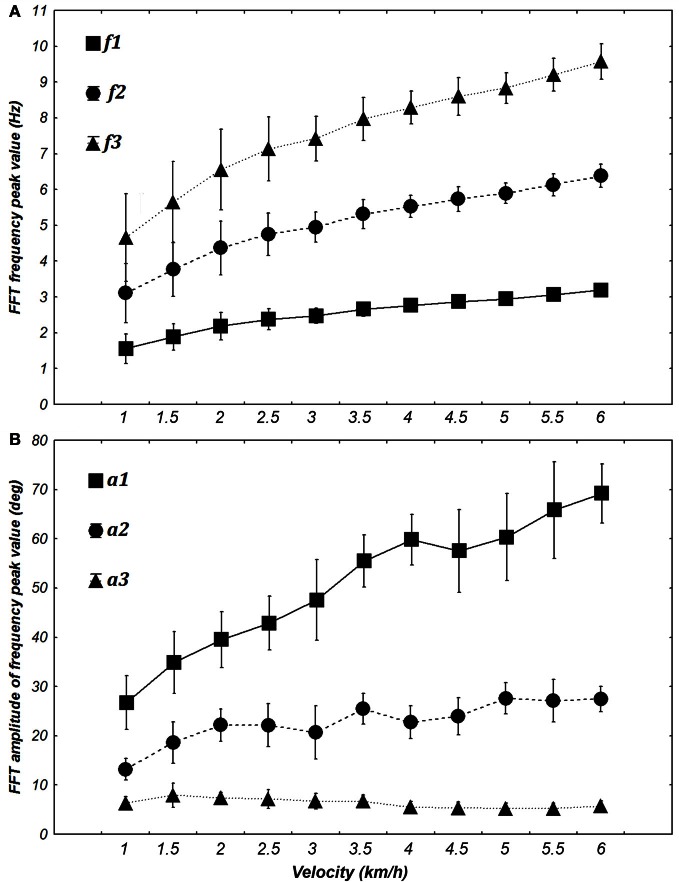
**Evolution of the frequency (A) and amplitude (B) of the first three harmonics when transforming elevation angles into the time frequency domain (FFT) at different walking velocities [from 1 to 6 km/h (0.28 to 1.67 m/s)].** The FFT peaks of each angle are averaged for each velocity for both frequency and amplitude. Squares, circles, and triangles represent the mean value for 7 subjects and whiskers represent the 95% confidence interval.

ANOVA shows significant changes in amplitude [*F*_(10, 60)_ = 31.351, *p* < 0.0001] and frequency [*F*_(10, 60)_ = 69.276, *p* < 0.0001] with an increase in velocity. *Post-hoc* analyses revealed an increase in *f*1, *f*2, *a*1, *a*2, *a*3 and a decrease of *f*3 with an increase in the velocity. These significant differences justified their use for building specific sine waves for different walking velocities (Figure 6).

**Figure 7:**
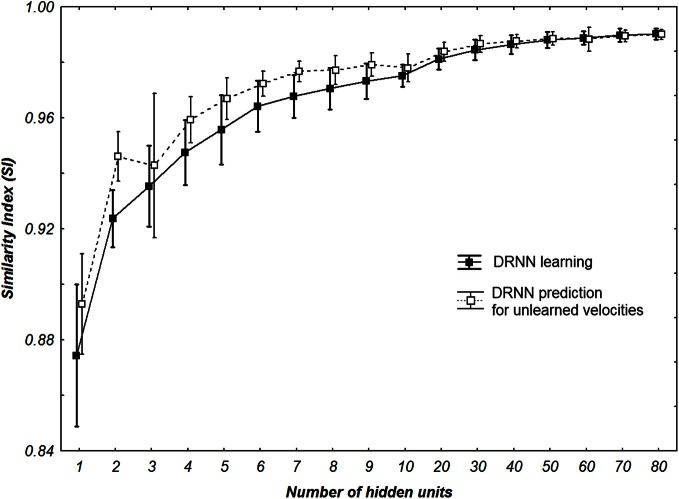
***SI* values of all multi-patterns learning and prediction for all participants with different number of hidden neurons.** Whiskers correspond to 95% confidence interval.

Concerning the performance of the DRNN outputs, we wanted to verify if the *SI* value applied for the best networks was different for learning and prediction. Additionally we wanted to statistically check if the number of hidden units of the networks increases the *SI* value (Figure 7). The Kolmogorov–Smirnov did not verify that the populations of *SI* values among the learning (*D* = 0.18467, *p* < 0.01) or the prediction (*D* = 0.20684, *p* < 0.01) were normal. Thus, to compare the *SI* values between learning and prediction process we chose to use a sign test as the structure of the network (weights and time constant) were the same. This test reveals no significant differences in *SI* values between the two populations except when the network contains 1, 2, 4, 5, 6, and 7 neurons. Moreover we use a Friedman test to analyze *SI* values (dependent variable) according to the number of hidden units (independent variables) of the network. There is an effect of the number of hidden units to the *SI* both for learning [χ^2^_(16)_ = 111.5630, *p* < 0.0001] and for prediction [χ^2^_(16)_ = 109.3721, *p* < 0.0001].

We also analyze the output of the DRNN for specific velocities where the hidden layer was the biggest with 80 units. A Kolmogorov–Smirnov run together with learning and prediction values did not verify that the populations of *SI* was normal (*D* = 0.18342, *p* < 0.05).

**Figure 8:**
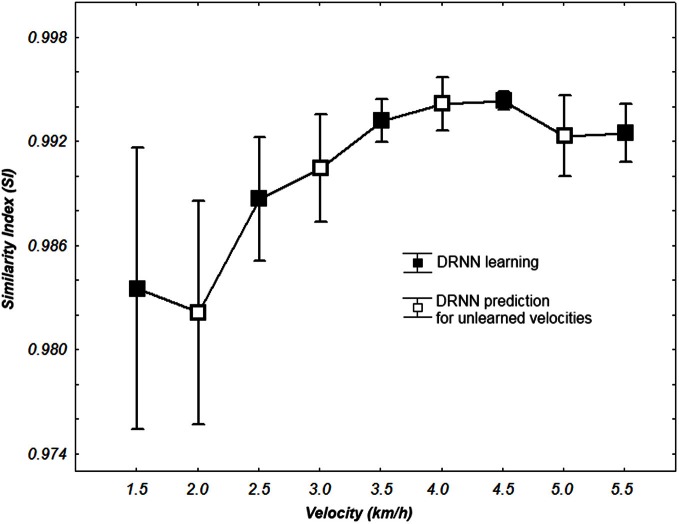
***SI* values for learning and prediction with a 80 hidden units network according to walking velocity.** Whiskers correspond to 95% confidence interval.

**Figure 9:**
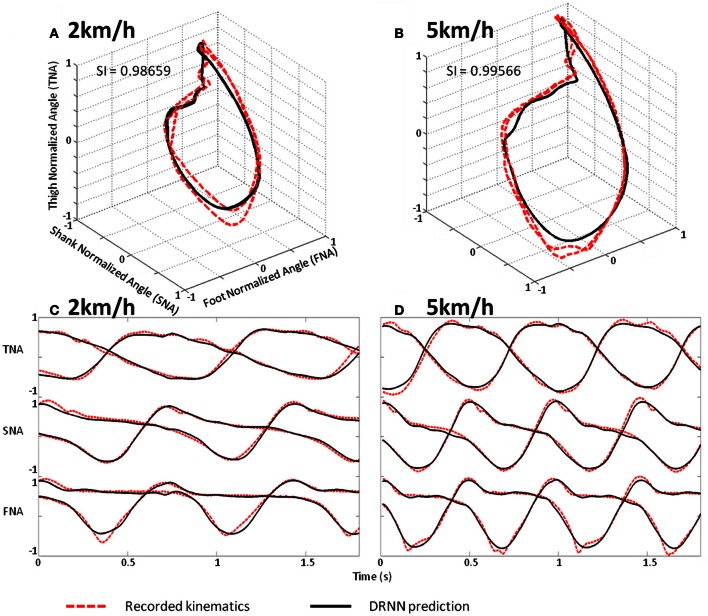
**Prediction of intermediate velocities in one subject. (A)** Planar covariation of the normalized thigh, shank, and foot for one participant walking at 2 km/h. **(B)** Planar covariation of the normalized thigh, shank, and foot for the same participant walking at 5 km/h. **(C)** Kinematics of walking of the normalized thigh, shank, and foot for the same participant walking at 2 km/h. **(D)** Kinematics of walking of the normalized thigh, shank, and foot for the same participant walking at 5 km/h. In each subplot the red dotted line corresponds to the experimental data whereas black thick line corresponds to the output of the DRNN.

We use a Friedman test to analyze *SI* values (dependent variable) according to the velocity (independent variables) of the network. There is an effect of the velocity to the *SI* [χ^2^_(8)_ = 30.22, *p* = 0.00019] (Figure 8). A *post-hoc* analysis at 0.05 significance level reveals that *SI* values of 4.5 km/h were different from 1.5 and 2 km/h. *SI* values of 4 km/h were also different from 2 km/h. An example of prediction of intermediate velocities in one subject is illustrated in (Figure 9).

### DRNN structures from Experiment 2

#### Weight distribution analysis

**Figure 10:**
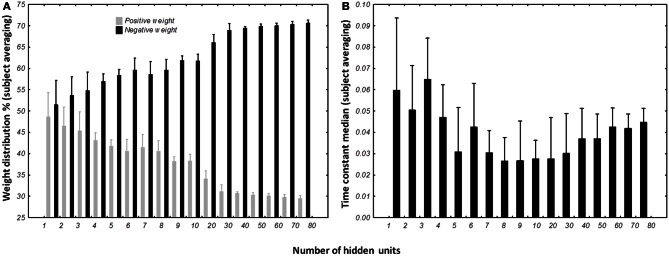
**Distribution of weights (A) and time constants (B) among the DRRN structures.** The horizontal axis represents the number of hidden neurons and vertical the percentage of population. The bars represent the mean distribution among the best DRNN for each subject. Whiskers represent the 95% confidence intervals.

The percentage of positive and negative weights was calculated for each best network per subject per condition. We wanted to verify if the distribution of the positive and negative weights were different (Figure 10A). The Kolmogorov–Smirnov test did not verify that the distribution of positive weights were normal (*D* = 0.15022, *p* < 0.01) nor the distribution of negative weights (*D* = 0.15022, *p* < 0.01). We then used a non-parametric sign-test to compare the two-distribution as the two samples were dependent. Regardless of the number of hidden units, the test shows a difference between the distributions of the two populations (*Z* = 9.482, *p* < 0.0001). When the number of units in the test were included, it appears that the population of negative weights is higher than population of positive weights when the network possesses more than 3 hidden units (for 4 hidden units, *Z* = 2.268, *p* = 0.023342). When the structure of the DRNN reaches 80 hidden neurons, 70.6 ± 0.84% of the synapses are negative.

#### Time constant distribution analysis

The distribution of the time constant was represented by the median of neuronal time constants of the best networks per subject and number of hidden units (Figure 10B). The Kolmogorov–Smirnov displayed a normal distribution of the time constant median (*D* = 0.09110, *p* > 0.20). ANOVA with repeated measures was designed with the number of hidden units as independent variable and time constant median as dependent variable. ANOVA analysis shows an interesting effect of the number of hidden neurons [*F*_(16, 96)_ = 3.6245, *p* < 0.0001]. Overall, *post-hoc* analysis revealed that the distribution of the time constants were different when the network was small (less than 5 hidden units). It also reveals that the networks with 80 hidden neurons were differently distributed than medium-sized network (8–10 hidden neurons).

## Discussion

### Main finding

We show here that a fully connected recurrent neural network is able to reproduce human walking pattern based on oscillatory properties of kinematics. Although, this network is a black box model without prewired structure mimicking a physiological CPG, its actual performance allows direct comparison with CPG dedicated structure and related algorithm (Duvinage et al., 2012a). Moreover, by the inherent input-output mapping, the DRNN models not only the CPG but also neural feedback pathways and the musculoskeletal system. For simplicity, we consider this neural network as “CPG-like structure” here. We proved that the DRNN is capable of generating the kinematics as elevation angles pattern of walking for both limbs (six degrees of freedom) from simple oscillations corresponding to the three main harmonics of the walking kinematics. Moreover, by modulating those frequencies and tuning them in amplitude as input, the DRNN was able to learn and reliably predict walking kinematics at different velocities (Figure 9). After this appropriate learning the DRNN can thus be considered as a CPG-like structure that would continuously receive oscillation inputs to produce the relevant elevation patterns of the six leg segments. Another interesting result is observed when looking at the structure of best CPGs obtained after learning. Hence, it appears that all of them contain a major part of negative connection weight between units.

### Limitations of the present approach

Obviously, there are infinite different ways to train the DRNN and this is a strength of this approach. However, it implies a corollary limitation as not all possibilities could be tested in the present study. For example, in order to better document its generalization ability, the model could be trained for a defined low range of velocities, e.g., from 1 to 4 km/h, and then tested with unlearned oscillation input corresponding to higher velocities. A reverse procedure could also be made, i.e., from faster training to slower predictions. Furthermore, inter-subjects generalization has not been studied in the present investigation. The actual usefulness of performing this would largely depend on the basic or application purposes. Another limitation of the present work is the lack of feedback testing, which necessitates a priori identification of a reliable signal and a new operational strategy for learning. Future work will address these aspects specifically.

### Neurophysiological similarity between modeled CPG and CPG in humans and other mammals

The understanding of CPG mechanisms remains central in locomotion study (Grillner, 2006; Kiehn, 2006; Rossignol and Frigon, 2011). The CPG is a spinal network of neurons capable of generating a rhythmic pattern of alternate activities between flexor and extensor motoneurons on the same side with reciprocal activation of homologous motoneurons in the contralateral limb. This intrinsic spinal circuitry has been well described in many invertebrate and vertebrate animals, and is highly conserved even in humans, where greater cortical control of spinal modules is required working in conjunction with sensory feedback (Calancie et al., 1994; Bussel et al., 1996; Duysens and Van de Crommert, 1998; Drew et al., 2002; Rossignol et al., 2009). The unique characteristics of human walking probably reflect a complex neural mechanism responsible for pattern production. It is therefore difficult to directly extend experimental findings obtained in quadruped animals to human walking (Barbeau et al., 1998; Capaday, 2002).

The fact that some patients with incomplete spinal injury can move their legs in a rhythmic fashion (Dietz et al., 1995) and that the primary sensorimotor cortex provides oscillatory commands toward the spine during walking (La Fougère et al., 2010) motivates new experiments in which different types of oscillatory signals could be used as input to the CPG-like DRNN. In this context, recent studies have showed EEG oscillations in relation to the gait cycle phase including event-related spectral perturbation in the alpha-beta and gamma bands (Gwin et al., 2011; Haefeli et al., 2011; Cheron et al., 2012; Wagner et al., 2012).

These results are consistent with a top down control of locomotion (Capaday, 2002) and demonstrate the feasibility of extracting EEG signals from the sensorimotor cortex controlling the contralateral foot placement during walking. Although the distinction between the brain signals directly linked to the motor commands and those related to the treatment of multiple sensory signals is a hard task. In this context, Petersen et al. (2012) have found evidence of synchrony in the frequency domain between the primary motor cortex and the tibialis anterior muscle prior to heel strike during the swing phase of walking signifying that rhythmical cortical activity is transmitted via the corticospinal tract to the active muscles. Additionally Wagner et al. (2012), showed a significant difference in the alpha (8–12 Hz) and beta (18–21 Hz) rhythm recorded over the central midline area between passive and active walking with exoskeleton. The role played by specific oscillations related to the initiation and control of human locomotion coming from supraspinal structure was recently demonstrated by local field potential recordings performed in the pedunculopontine nucleus in parkinsonian patients during rest and unconstrained walking (Thevathasan et al., 2012). Alpha oscillation recorded in the caudal part of this nucleus is correlated with gait speed and permits to suppress “task irrelevant” distraction for improving gait performance. Moreover, these authors showed that gait freezing of parkinsonian patient was associated with the attenuation of these alpha waves.

Consistent with this aspect of gait physiology, in our model, input sine waves are sufficient to predict successful output with the DRNN and offer the possibility to mimic such type of supraspinal oscillatory input. It could be advanced that nonlinear mapping of sinusoidal oscillations to kinematic pattern should be realized by other mathematical functions, such as by a Taylor series, but such multi-dimensional approximation seems to be highly difficult to obtain and does not permit testing different network configurations mimicking biological organization such as the CPG. In the present study, we focused on input oscillation derived from the first three harmonics of kinematic signals, which were slower than the alpha frequency range. However, it could be possible to extract slower oscillation from alpha or beta derived signals (envelope) in order to activate the DRNN in our future work.

### Structural similarities of modeled CPG and neurophysiological CPGs in animals

It has been suggested that the biological CPG is considered to serve two basic functions: rhythm generation (RG) and pattern formation (PF).

Initially proposed by Perret and Cabelguen (1980), the idea that the biological CPG is composed of a rhythm and a pattern-amplitude generator is now widely accepted (Kriellaars et al., 1994; Guertin et al., 1995; Perreault et al., 1995; Grillner, 2006; Kiehn, 2006; Talpalar et al., 2011) and paved the way to more complex models of multi-level CPG (McCrea and Rybak, 2008, see below).

It is well-recognized that rhythm generating networks can be realized by means of (1) pacemaker neurons with intrinsic membrane properties such as those described in the stomatogastric ganglion of crustaceans or in the mammalian thalamus (Steriade and Llinás, 1988) or (2) most simplistic neurons without intrinsic pacemaker properties but interacting with inhibitory synapses for producing oscillation as emergent properties of this neuronal population (Geisler et al., 2005). Both neuronal systems thus present the fundamental ability to oscillate. Firstly described in the tadpole and lamprey CPGs, glutamatergic excitatory neurons distributed along the cord (Grillner, 2003) assume the function of rhythmic generator by driving motor neurons and other ipsilateral and commissural inhibitory neurons coordinating the different CPG modules. By blocking the inhibitory networking in the lamprey and also in rodent and cat, many authors (see Kiehn, 2006 for a review) have demonstrated that the glutamatergic burst neurons are the generators of the CPG rhythm.

In addition to intrinsic RG properties, the walking CPGs need to integrate the ipsilateral coordination of flexors and extensors across the same or different joints in a limb and perform inter-limbs coordination. It has been proposed (Zhong et al., 2012) that a subpopulation of neuronal CPG that drives extensor activity is tonically active and is regulated via inhibitory interactions with another CPG rhythmic structure responsible for flexors activity in the same hemicord. This assumption may explain why, during experimental recordings on the neonatal mouse isolated spinal cord, spontaneous deletions of extensor activity do not perturb rhythmic flexor activity. Thus, the inhibitory interneurons play a major role in the temporal sculpting and coordination of the CPG units. The interneurons and the Renshaw cells are involved in this function and in the regulation of walking speed. In addition, the left-right coordination is assumed by a complex network of excitatory and inhibitory commissural interneurons acting on both motor neurons and inhibitory interneurons of the contralateral side (Kiehn, 2006). Interestingly, we have shown that a great percentage of artificial neurons became inhibitory neurons (negative synaptic weight) when the number of neurons progressively increases in the DRNN structure. In this context, it was recently demonstrated in awake mice that the spiking activity of inhibitory neurons of the barrel cortex is organized in order to balance excitation and prevent explosive activity in the recurrently connected cortical microcircuit (Gentet et al., 2010). This physiological mechanism can also be proposed in the present case of the emergent structure of artificial DRNN circuit. Another, not exclusive explanation can reside in the prevalence of inhibitory recurrent connections for producing network oscillation (Geisler et al., 2005; Wildie and Shanahan, 2011). In their review, Nishimaru and Kakizaki (2009) have proposed that inhibitory interneurons play a major role in the CPG of rodent spinal cord. The interneurons are likely to control the bursting of motor neurons during locomotion and it appears that the synaptic transmission mediated by glycine and GABA shifts from excitatory to inhibitory during the prenatal period. It was recently demonstrated that in the absence of glutamatergic synaptic transmission, the flexor-extensor alternation appears to be generated by the inhibitory interneurons, mediating reciprocal inhibition from muscle proprioceptors to antagonist motor neurons (Talpalar et al., 2011).

The present artificial model does not pretend to mimic the complexity of the CPG structure. Instead, it presents a highly simplified recurrent organization from which CPG-like dynamic function emerges, following appropriate learning. Sinusoidal inputs serve as temporal referent to produce rhythmic angles patterns. This model could correspond to the RG structure described previously as a higher order structure that determines rhythmic output of the system (McCrea and Rybak, 2008; Zhong et al., 2012) since sine waves are transformed into kinematics. Another, lower order structure responsible for the phasing and intensity coordination (McCrea and Rybak, 2008; Zhong et al., 2012) could be assumed by another model of DRNN transforming theoretical kinematics into practical muscle orders. We have already studied such relation where EMG signals from walking where used to predict kinematics (Cheron et al., 2003). To conclude to this point, we propose that the two model driving two specific DRNNs (one for producing elevation angles from sine waves and one producing muscular patterns from elevation angles during walking) could act as a complementary top-down pathway to produce adequate coordinated patterns as it has been proposed to model the locomotion in spinal mouse (Zhong et al., 2012).

The present results can also be discussed in the light of the electrical stimulations performed in the MLR) inducing locomotor behavior in decerebrate cat (Shik et al., 1969) or in lamprey (McClellan and Grillner, 1984). In mice, prolonged rhythmic stimulation on the midline of the caudal hindbrain or the ventral spinal cord (C1–C4) induces a stable locomotor activity (Talpalar et al., 2011). Typically, low-frequency stimulation leads to slow-frequency movements and inversely fast-frequency stimulation leads to fast-frequency movements. Our model is in accordance with this physiological behavior, when amplitude of the artificial sine wave inputs increases, the amplitude of stepping increases as well, leading to a change in walking velocities (Figure 6B). In terms of neurological development, there is some evidence for the existence of CPG very early in CNS maturation (Yang et al., 1998; Dominici et al., 2011). Neonatal, so-called “infant” stepping has been ascribed to similar EMG patterns activity in different directions inducing stereotyped yet non-functional walking patterns (Lamb and Yang, 2000). This leads the authors to conclude that the same CPG controls different stepping in human infants in contrast with some studies in adults (Thorstensson, 1986; Grasso et al., 1998). Interestingly, we found that DRNN with only four hidden artificial neurons can generate walking pattern, whereas at least 50 hidden neurons are the prerequisite to generate accurate movements (Figure 7). Obviously, recruitment and training of such high numbers of neurons requires long computational time. For example, with a 4 hidden units DRNN structure, the learning process lasts about 5 min based on our computer performance while 160 min are necessary for a DRNN containing 80 units (Figure 4).

### Perspectives

Such a tool can be used to produce gait kinematics in numerous and various applications. For rehabilitation it can be used to train people for recovering a walking pattern corresponding to their physical characteristics by training with an appropriate feedback. Specifically dedicated DRNN based on the proper dynamics of participants could be used for medical applications such as in prosthesis and exoskeleton control (Cheron et al., 2012). It can also be integrated in BCI applications in which higher order commands can be used, e.g., from steady state visual or somatosensory evoked potentials (Cheron et al., 2012) or P300 (Castermans et al., 2011; Duvinage et al., 2012b). This neuronal avenue might lead to the decoding of higher neuronal commands that govern CPG mechanisms. Since these CPG can be trained using specific sinusoidal frequency signals, it might be possible to extract this type of signals from specific EEG rhythms since the brain itself is an effective machine for producing oscillations (Buzsáki and Draguhn, 2004). One of the strengths of this approach is that it is not necessary to determine in advance the topology and the timing sequences between the artificial neurons. This contrasts with other CPGs, such as a recently developed ones (Duvinage et al., 2011) based on coupled oscillators (Righetti and Ijspeert, 2006), where adjustment of intrinsic parameters by optimization techniques was necessary.

In future studies, by introducing an informational delay (Draye et al., 1997) or an artificial distance based on a Gaussian factor modulating the weights between the different neurons (Draye et al., 2002), it will be possible to analyze the self-tailored organization of the links between neurons and the possible emergence of specific topologies. In this case it will also be possible to select different modular architectures of the DRNN.

### Conflict of interest statement

The authors declare that the research was conducted in the absence of any commercial or financial relationships that could be construed as a potential conflict of interest.
